# ITRAQ-based quantitative proteomics analysis of forest musk deer with pneumonia

**DOI:** 10.3389/fvets.2022.1012276

**Published:** 2022-10-26

**Authors:** Jie Tang, Lijuan Suo, Feiran Li, Chao Yang, Kun Bian, Yan Wang

**Affiliations:** Shaanxi Key Laboratory for Animal Conservation, Shaanxi Institute of Zoology, Xi'an, China

**Keywords:** forest musk deer, pneumonia, proteomics, bacterium, immunity

## Abstract

Pneumonia can seriously threaten the life of forest musk deer (FMD, an endangered species). To gain a comprehensive understanding of pneumonia pathogenesis in FMD, iTRAQ-based proteomics analysis was performed in diseased (Pne group) lung tissues of FMD that died of pneumonia and normal lung tissues (Ctrl group) of FMD that died from fighting against each other. Results showed that 355 proteins were differentially expressed (fold change ≥ 1.2 and adjusted *P*-value < 0.05) in Pne vs. Ctrl. GO/KEGG annotation and enrichment analyses showed that dysregulated proteins might play vital roles in bacterial infection and immunity. Given the close association between bacterial infection and pneumonia, 32 dysregulated proteins related to *Staphylococcus aureus* infection, bacterial invasion of epithelial cells, and pathogenic *Escherichia coli* infection were screened out. Among these 32 proteins, 13 proteins were mapped to the bovine genome. Given the close phylogenetic relationships of FMD and bovine, the protein-protein interaction networks of the above-mentioned 13 proteins were constructed by the String database. Based on the node degree analysis, 5 potential key proteins related to pneumonia-related bacterial infection in FMD were filtered out. Moreover, 85 dysregulated proteins related to the immune system process were identified given the tight connection between immune dysregulation and pneumonia pathogenesis. Additionally, 12 proteins that might function as crucial players in pneumonia-related immune response in FMD were screened out using the same experimental strategies described above. In conclusion, some vital proteins, biological processes, and pathways in pneumonia development were identified in FMD.

## Introduction

Forest musk deer (FMD, *Moschus berezovskii*) is a type of mammal that mainly lives in the alpine forests in China and Vietnam (1, 2). The populations of FMD are sharply declined since the 1950s due to habitat destruction/degradation and massive hunting for their musk (Moschus) (3, 4). Musk is the dried secretion from the musk sac gland of male musk deer, such as *Moschus berezovskii Flerov, Moschus moschiferus Linnaeus*, and *Moschus sifanicus Przewalski* (5, 6). Musk is a superior component in perfume and is believed to have potential therapeutic values for multiple diseases such as cancers, strokes, and heart diseases in the traditional Asian medicine industry (6, 7). This species has been listed in the First-Class National Protected Animal List of China and is protected under the Chinese Wild Animal Protection Law (4, 8). Moreover, FMD is the major species of musk deer that can be reared artificially in special farms under the support of the Chinese government, which contributes to the growth of the population, reduction of poaching behaviors, and better utilization of FMD resources (9, 10).

The increase in the population of FMD is also limited by some fatal diseases including pneumonia (11–13). Pneumonia can be caused by multiple pathogens including bacteria (14–16). However, previous research on pneumonia mainly focused on the isolation, identification, and genome analysis of pathogens in FMD (12, 17, 18). To reduce the risk and harm of pneumonia for the health and life of FMD, it is imperative to have an in-depth insight into the molecular mechanisms underlying pneumonia development and identify key molecules or pathways related to pneumonia pathogenesis.

Recently, mass spectrometry (MS)-based proteomics has attracted much attention from researchers because proteins are responsible for most biological functions and proteomics can simultaneously capture and quantify thousands of proteins rather than RNAs in a cost-effective manner (19, 20). Isobaric tag for relative and absolute quantification (iTRAQ), an isotope labeling strategy, has been widely used in proteomics studies needing relative quantification due to the multiple advantages such as multiplexing capacity, reproducibility, easy operation, and flexibility (21–23). The combination of iTRAQ and MS-based proteomics technologies and bioinformatics analytical methods have emerged as a powerful strategy for identifying vital proteins related to disease pathogenesis, comprehensively understanding protein roles and basic biological functions, and deciphering complicated molecular mechanisms underlying disease development in multiple animals (24–26).

However, to our knowledge, there is no proteomics data to explore the pathogenesis of pneumonia in FMD to date. To build up a general and comprehensive understanding of pneumonia pathogenesis, the iTRAQ-based LC-MS/MS technique was used to explore the proteomics alterations in diseased lung tissues of FMD that died of pneumonia than in normal lung tissues of FMD died from fighting against each other. Moreover, some genes/proteins, biological processes, and signaling pathways that might play vital roles in pneumonia progression were screened out based on differential expression, annotation, enrichment, and protein-protein interaction analysis.

## Materials and methods

### Animal samples

Forest musk deer are reared in the Shaanxi Institute of Zoology (China). The Animal breeding area (34.210832°N, 106.902117°E) is located in Fengxian, Southwest of BaoJi City, Shaanxi Province, China, a region of Qinling mountain at an altitude of 1,500 m. Diseased lung tissues were obtained from 3 adult FMD (2♂1♀, 4.5 years old) that died of pneumonia. Normal lung tissues were obtained from 3 adult FMD (♂, 3.5 years old) that died from fighting against each other. Tissue samples with a weight of no <200 mg were stored at −80°C. The study was approved by the Academic Committee of Shaanxi Institute of Zoology with Ethical Approval No.: 20210327001.

### Histological analysis

The tissues mentioned above were fixed in a PBS buffered formaldehyde solution for 48 h. After routine dehydration and transparency, sectioned at a thickness of 4 μm and stained with Eosin Staining Kit (Beyotime, Shanghai, China) following the protocols of the manufacturer, and examined by light microscopy.

### ITRAQ-based proteomics analysis

ITRAQ-based proteomics analysis was performed as previously described (27, 28). The detailed experimental procedures of proteomics analysis including sample preparation, iTRAQ labeling and fractionation, LC-MS/MS analysis, and data analysis were shown in Supplementary file 1. Briefly, tissue samples were ground to a fine powder in liquid nitrogen and then lysed using the protein lysis buffer [7M Urea/4% SDS/2M Thiourea/40 mM Tris-HCl (pH 8.5)] supplemented with 2 mM EDTA and 1mM phenylmethylsulfonyl fluoride. Samples were labeled using the iTRAQ Reagent-8 plex Multiplex Kit (SCIEX, Framingham, MA, USA) according to the protocols of the manufacturer, in which only 6 channels were used in our project. The information of sample-corresponding channels was shown in Table 1. Pne and Ctrl groups represented diseased and normal lung tissue groups, respectively. LC-MS/MS analysis was carried out on TripleTOF 5600+ mass spectrometry (SCIEX) coupled with an EksigentnanoLC system (SCIEX). Raw data analysis was performed using the Protein Pilot Software (version 4.5, SCIEX). The raw MS/MS file data were searched against the PR1-19060015_pep. fasta (containing 24,352 sequences). Proteins were regarded to be significantly differentially expressed when fold-change ≥ 1.2 and adjusted *P*-value < 0.05.

**Table 1:** Sample-corresponding iTRAQ channels.

**Sample groups**	**Sample label**	**Channel**
Disease-1	Pne_1	113
Disease-2	Pne_2	114
Disease-3	Pne_3	117
Normal-1	Ctrl_1	118
Normal-2	Ctrl_2	119
Normal-3	Ctrl_3	121

### Bioinformatics and annotations

To determine the biological and functional properties of all the identified proteins, the identified protein sequences were mapped with those in the Swiss-Prot database using BLASTP. In addition, a homology search was performed for the differentially expressed protein sequences using a localized NCBI blastp program against the NCBI non-redundant protein (NR) animal database. Moreover, the GO and KEGG annotation information of matched proteins was extracted. GO and KEGG pathway enrichment analysis was performed using the hypergeometric test. GO and KEGG pathway terms were considered to be significantly enriched at a *P*-value < 0.05. Protein-protein interaction (PPI) networks were constructed using the STRING database (version: 11.5) (https://cn.string-db.org/).

### qRT-PCR analysis for gene expression

Ten mRNAs were randomly selected for expression analysis by qRT-PCR to validate the data. The primer sequences are listed in Table 2. The GAPDH gene was used as the internal control. The total RNA was extracted from the Lung tissues with the RNAiso plus reagent (Takara, Dalian, China) following the manufacturer's protocols. The qRT-PCR was performed using SYBR Premix ExTaq (TaKaRa, Dalian, China) and a Thermal Cycler CFX96 Real Time-PCR detection system (Bio-Rad, Hercules, CA, USA) with the following parameters: 95 °C for 60 s; 40 cycles at 95 °C for 15 s; 60 °C for 30 s; and 72 °C 10 s. The concentration and purity of total RNA were measured using a GE Nanovue™ Spectrophotometer (GE Health care Biosciences, Pittsburgh, USA). cDNA was synthesized using the SYBR Prime Script™ RT Master Mix (Perfect Real Time) Kit (Takara, Dalian, China). The relative expression of each gene was calculated with the 2–ΔΔCt method. There were three biological sample replicates, and each biological sample replicates included three technical replicates.

**Table 2:** Primers used in quantitative real-time PCR analysis.

**Target gene**	**Primer**	**Sequence (5'to3')**
GAPDH	GAPDH-F	GGCACAGTCAAGGCAGAGAAC
	GAPDH-R	TACTCCGCACCAGCATCACC
Galectin-9	Galectin-9 F	CGGTTTGAAGAAGGCGGGTATG
	Galectin-9 R	AGATGGCGTTGAATTGGTAGAAGG
Coronin-1A	CORO1A-F	CACTTTGGATGAGGAGCAGAA
	CORO1A-R	TGGCTGGCTGTCCAAATAC
Annexin A6	ANXA6-F	AATGACACCTCTGGCGAATAC
	ANXA6-R	ACTGCACTAAGTTCCCACATC
Protein S100-A10	S100A10-F	TGCCGTCTCAAATGGAACA
	S100A10-R	TCCATGAGTACTCTCAGGTCTT
Moesin	MSN -F	AGAAGAGGTGGCAAGAATACAC
	MSN -R	TTCCAGGATGTCTGGCTCTA
Envoplakin	EVPL -F	TTCCAGGATGTCTGGCTCTA
	EVPL -R	GTAGGTTCTTGCACTCCCTATG
Platelet endothelial cell adhesion molecule	PECAM1-F	GAGTATGAGGTGTGGGTGAAAG
	PECAM1-R	CTGGGACAGAACAGTTGACTAC
Integrin beta-1	ITGB1-F	AGGCCACTGTTCATGTTGTAG
	ITGB1-R	CAGCAATGCAAGGCCAATAAG
CD177	CD177-F	CTACTGAACCTACCCAAGACAAG
	CD177-R	GCAGAGGTGATGTTGATGAGTA
Collectin-12	COLEC12-F	CAACTCAGAACTCTCCACCTTC
	COLEC12-R	TGGCCAAAGCGGAGTTATT

## Results

### Histological observation of lung tissue

**Figure 1:**
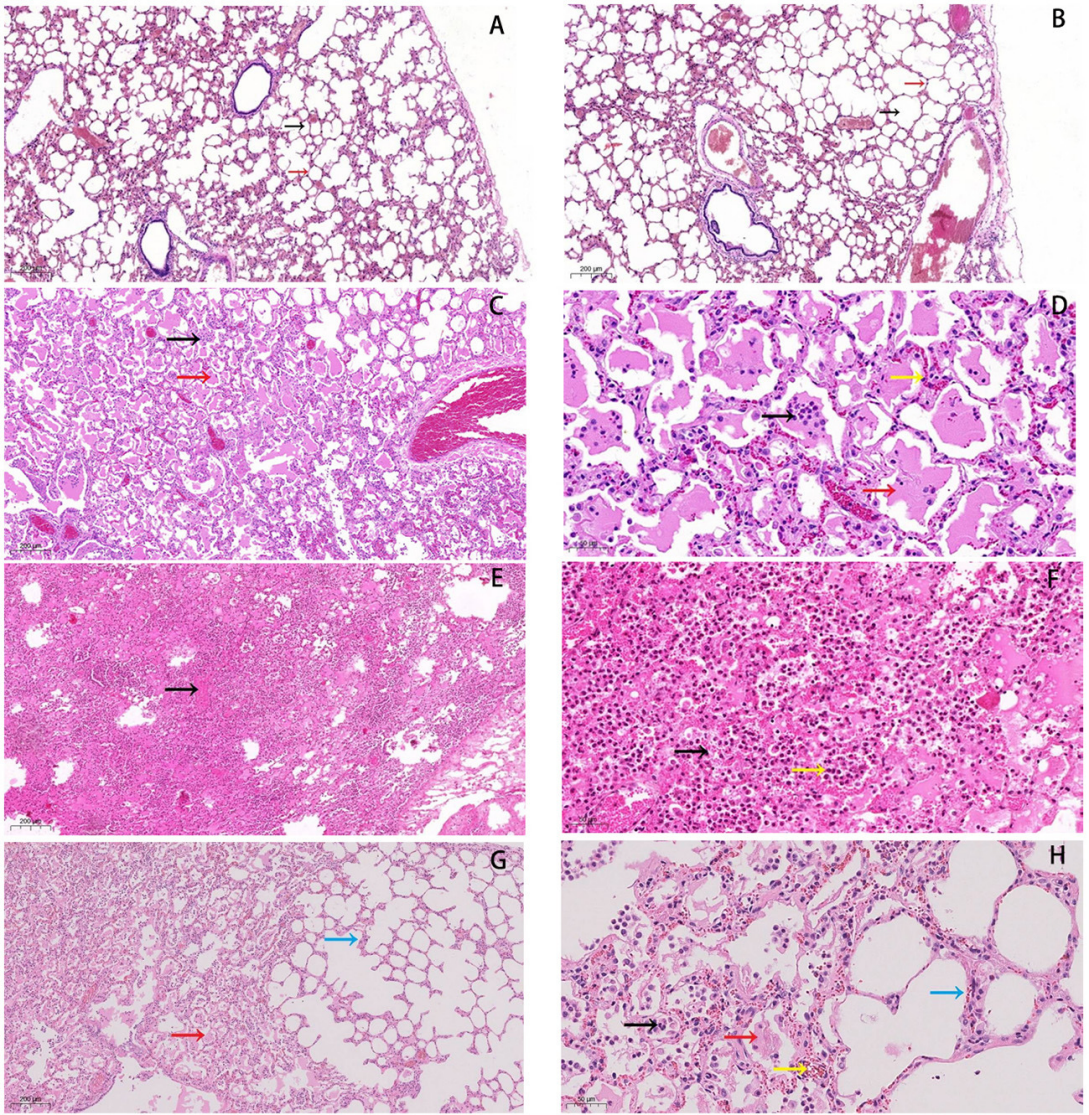
Histopathological changes in lungs of forest musk deer. **(A,B)** normal lung tissue in the control group, HE staining of normal tissues was performed (5×). The black arrow indicates the alveolar cavity, the red arrow points to the alveolar wall. **(C,D)** Inflammatory cells (black arrow) and exudate can be seen in the alveolar cavity (red arrow), and the capillaries in the alveolar wall are hyperemic (yellow arrow). **(E,F)** Numerous broken neutrophils (yellow arrow) exuded from the alveolar cavity, bleeding obvious (black arrow). **(G,H)** Red blood cell (yellow arrow), inflammatory cell (black arrow) and fibrin exudates (red arrow) present in alveolar lumen, and lung interstitium is widened (blue arrow). Bars: **(B,D,F,H)** 50 μm, Bars: **(A,C,E,G)** 200 μm, (magnification, 5.0×, 20.0×).

Histological analysis showed that the alveolar cavity has inflammatory cell exudates and the alveolar wall capillary hyperemia in the pneumonia group (Figures 1C,D). Numerous broken neutrophils were exuded from the alveolar cavity and obvious bleeding was noticed in the pneumonia group (Figures 1E,F). And, the most notable pathological changes were interstitial pneumonia and hemorrhagic pneumonia in the pneumonia group (Figures 1G,H). Moreover, red blood cell, inflammatory cell, and fibrin exudate were present in the alveolar lumen, and the lung interstitium was widened in the pneumonia group (Figures 1G,H).

### Identification of differentially expressed proteins

**Figure 2:**
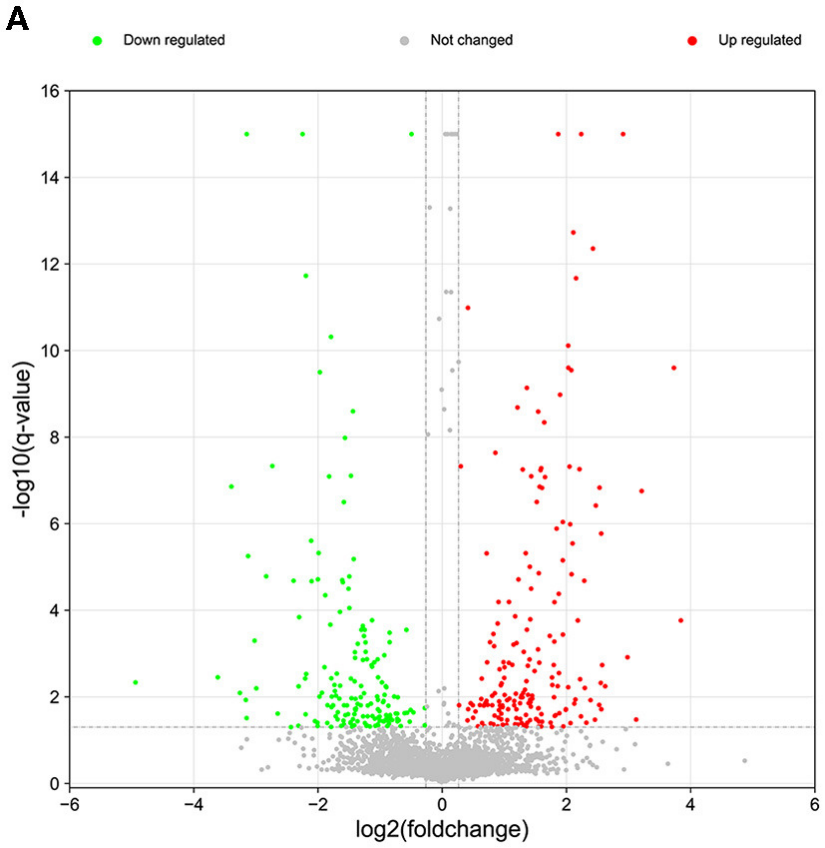
The volcano plot of differentially expressed and unchanged proteins. Red dots represent the up-regulated proteins and green dots denote the down-regulated proteins.

In our proteomics analysis, 355 proteins (169 down-regulated and 186 up-regulated) were found to be differentially expressed (up-regulated ratio ≥ 1.2 or down-regulated ratio ≤ 0.83; adjusted *P*-value < 0.05) in the diseased lung tissues of FMDs who died of pneumonia compared to the normal lung tissue group (Figure 2; Supplementary Table 1). The volcano plot of differentially expressed proteins was shown in Figure 2. Among these differentially expressed proteins, 158 proteins were annotated in the bovine Swiss-Prot database.

### GO and KEGG annotation analysis of differentially expressed proteins

**Figure 3:**
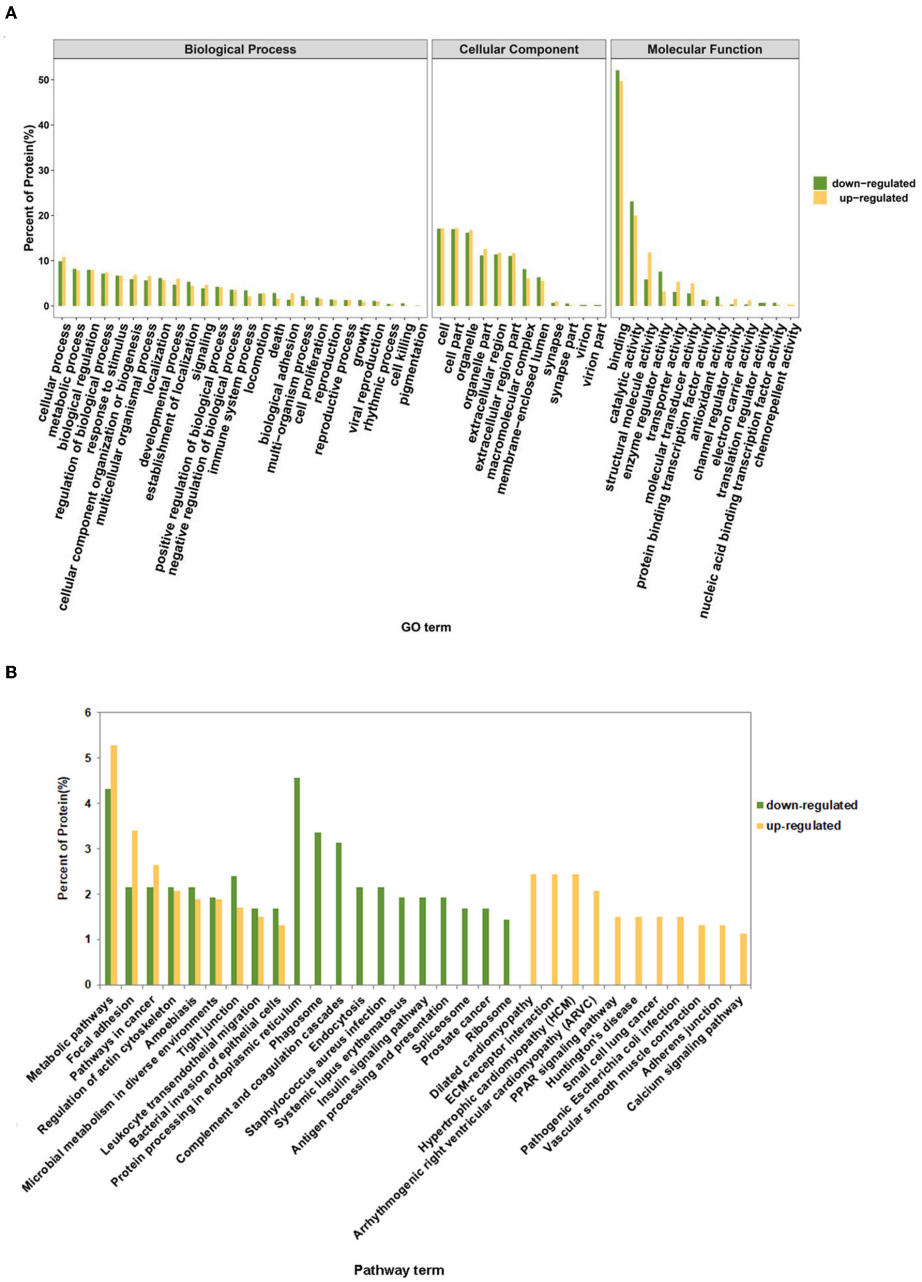
GO and KEGG annotation analysis of up-regulated and down-regulated proteins. **(A)** The percentage of up-regulated and down-regulated proteins in each GO term. **(B)** The percentage of up-regulated and down-regulated proteins in the top 20 annotated KEGG pathway terms.

To screen out key proteins related to the pathogenesis of pneumonia, the sequences of differentially expressed proteins were compared against the NCBI NR database using the NCBI-BLAST. These differentially expressed proteins were also annotated by comparisons against the GO and KEGG databases. Based on the principle of sequence similarity, proteins with similar sequences have similar functions. GO annotation analysis revealed that most of the down-regulated and up-regulated proteins were involved in the regulation of biological processes such as cellular process, metabolic process, biological regulation, response to stimulus, and cellular component organization or biogenesis (Figure 3A; Supplementary Table 2). Also, many differentially expressed proteins were implicated in the immune system process, death, locomotion, cell proliferation, biological adhesion, and growth (Figure 3A; Supplementary Table 2). Moreover, KEGG pathway annotation analysis showed that most up-regulated and down-regulated proteins played crucial roles in the pathways related to focal adhesion, phagosome, microbial metabolism in diverse environments, leukocyte transendothelial migration, bacterial invasion of epithelial cells, endocytosis, *Staphylococcus aureus* infection, and pathogenic *Escherichia coli* infection (Supplementary Tables 3, 4). The statistics of the top 20 KEGG pathways of up-regulated and down-regulated proteins were shown in Figure 3B.

### GO and KEGG enrichment analysis of differentially expressed proteins

**Figure 4:**
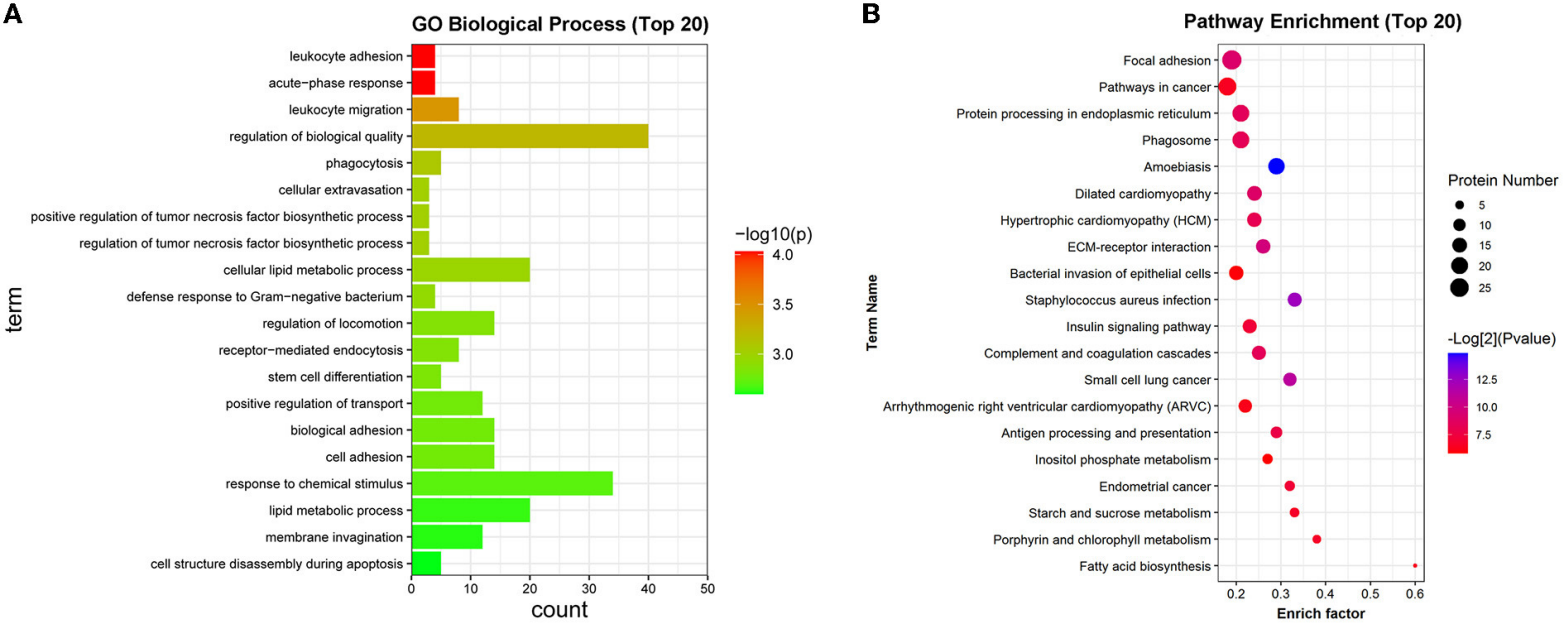
GO and KEGG enrichment analysis of differentially expressed proteins. **(A)** The top 20 GO biological process terms that were significantly enriched by the differentially expressed proteins. **(B)** The top 20 KEGG pathways that were significantly enriched by the differentially expressed proteins.

GO enrichment analysis showed that differentially expressed proteins were significantly enriched in biological processes such as acute-phase response, leukocyte adhesion, leukocyte migration, phagocytosis, regulation of tumor necrosis factor biosynthetic process, defense response to Gram-negative bacterium, regulation of locomotion, receptor-mediated endocytosis, cell structure disassembly during apoptosis, defense response to fungus (Supplementary Table 5). The top 20 GO biological process terms that were significantly enriched by differentially expressed genes were displayed in Figure 4A. KEGG enrichment analysis disclosed that differentially expressed proteins were significantly enriched in pathways related to *Staphylococcus aureus* infection, focal adhesion, complement and coagulation cascades, phagosome, antigen processing and presentation, bacterial invasion of epithelial cells, and pathogenic *Escherichia coli* infection (Supplementary Table 6). The top 20 KEGG pathways that were significantly enriched by the differentially expressed proteins were shown in Figure 4B.

### Screening and PPI network construction of dysregulated proteins related to bacterial infection

**Figure 5:**
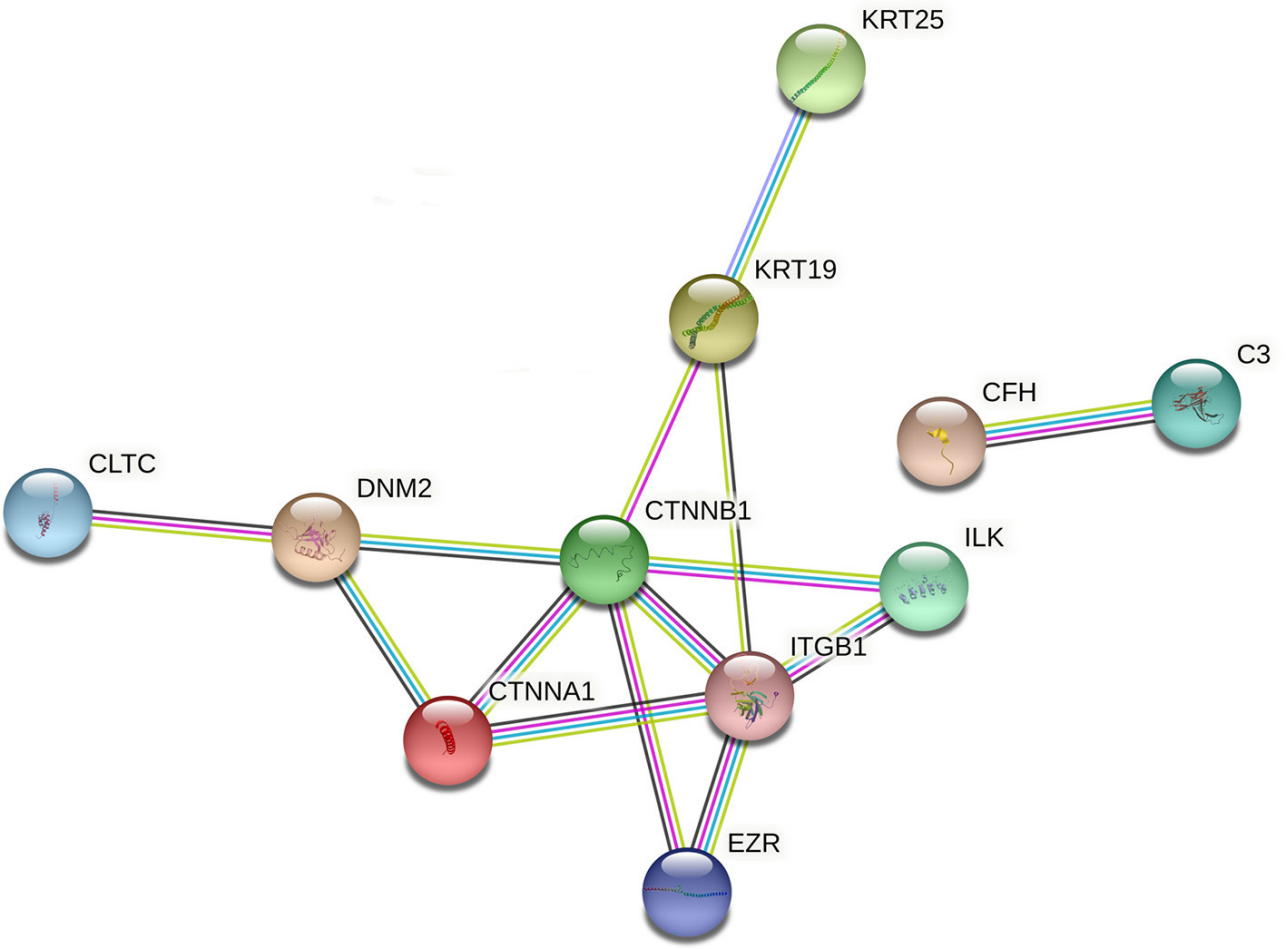
The PPI networks of differentially expressed proteins (species: bovine) related to bacterial infection. The darkturquoise and pink lines represented the interactions that were identified from curated databases and experimentally determined, respectively. The green, red, and blue lines represented the predicted interactions based on gene neighborhood, gene fusions, and gene co-occurrence relationships, respectively. The yellow-green, black, and purple lines denoted the potential interactions that were identified by textmining, co-expression, and protein homology. The full names of proteins in this figure were shown as follows: C3, complement C3; CFH, complement factor H, KRT19, keratin, type I cytoskeletal 19; KRT25, keratin, type I cytoskeletal 25; DNM2: dynamin-2; ILK: integrin-linked protein kinase; CLTC, clathrin heavy chain 1; CTNNA1, catenin alpha-1; ITGB1, integrin beta-1; CTNNB1, catenin beta-1; BCAM, basal cell adhesion molecule; TUBA4A, tubulin alpha-4A chain; EZR, ezrin.

Both KEGG annotation and enrichment outcomes suggested that pathways related to *Staphylococcus aureus* infection, bacterial invasion of epithelial cells, and pathogenic *Escherichia coli* infection might play vital roles in pneumonia progression. Given the close association between bacterial infection and pneumonia pathogenesis, dysregulated proteins in the above-mentioned pathways were filtered out based on KEGG annotation analysis. The information on these proteins was shown in Supplementary Table 7. As presented in Supplementary Table 7, 13 (9 down-regulated and 4 up-regulated), 14 (7 down-regulated and 7 up-regulated), or 12 (4 down-regulated and 8 up-regulated) differentially expressed proteins were identified to be implicated in *Staphylococcus aureus* infection, bacterial invasion of epithelial cells, or pathogenic *Escherichia coli* infection, respectively. The above-mentioned differentially expressed proteins related to bacterial infection (total number: 32) were integrated into Supplementary Table 8. The STRING database has been widely used to construct the PPI network and identify hub proteins in previous studies (29, 30). Prior phylogenetic tree analysis showed that FMD was a member of the suborder Ruminantia and order Artiodactyla with close phylogenetic relationships with four members of the family Bovidae (sheep, yak, cattle, and Tibetan antelope) (31). Also, a recent study showed that most FMD unigenes that were identified by *de novo* assembly of heart and musk gland transcriptomes were homologous with bovine genes (32). Given the close phylogenetic relationships of FMD and bovine, the PPI networks of filtered proteins were constructed based on the information of the organism species Bos taurus (bovine). Among filtered 32 differentially expressed proteins related to bacterial infection, 13 proteins were annotated in the bovine Swiss-Prot database (Supplementary Table 8). The PPI networks of the 13 proteins were constructed and displayed in Figure 4 (organism: Bos taurus, combined interaction score ≥ 0.4) (the solitary proteins had been removed from the network). The detailed interaction information of these 13 proteins was shown in Supplementary Table 9. The node degrees (number of interacted proteins) of proteins in the PPI networks (Figure 5) were exhibited in Supplementary Table 10. The node degree can be used to identify hub proteins in the PPI networks (33, 34). Results suggested that 5 proteins with greater node degrees [*i.e*. catenin beta-1 (CTNNB1), integrin beta-1 (ITGB1), catenin alpha-1 (CTNNA1), dynamin-2 (DNM2), Keratin, type I cytoskeletal 19 (KRT19)] might function as crucial players in pneumonia-related bacterial infection in FMD.

### Screening and PPI network construction of dysregulated proteins related to immunity

**Figure 6:**
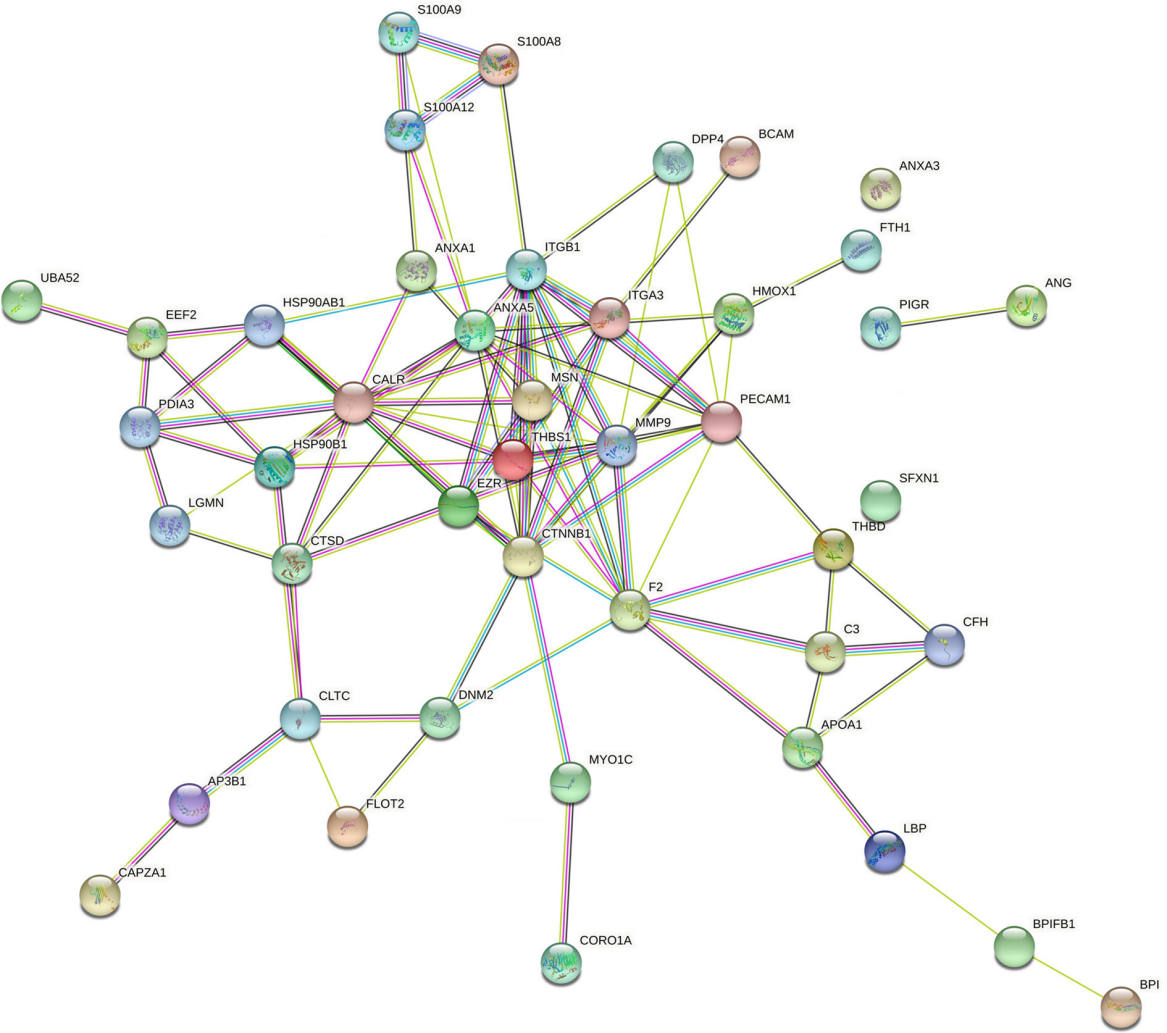
The PPI networks of differentially expressed proteins (species: bovine) related to immunity. The full names of the proteins in this figure were presented as below: CALR, calreticulin; LGALS9, galectin-9; C3, complement C3; EPB42, protein 4.2; HSP90B1, endoplasmin; CORO1A, coronin-1A; ANXA1, annexin A1; MMP9, matrix metalloproteinase-9; AP3B1,AP-3 complex subunit beta-1; BPI, bactericidal permeability-increasing protein; THBS1, thrombospondin-1; ANG1, angiogenin-1; S100A12, protein S100-A12; LBP, lipopolysaccharide-binding protein; ANXA5, annexin A5; S100A8, protein S100-A8; S100A9, protein S100-A9; HSP90AB1, heat shock protein HSP 90-beta; DHX9,ATP-dependent RNA helicase A; CAPZA1, f-actin-capping protein subunit alpha-1; GPI, glucose-6-phosphate isomerase; CLTC, clathrin heavy chain 1; DNM2,dynamin-2;F2, prothrombin; THBD, thrombomodulin (fragment); CFH, complement factor H; EEF2, elongation factor 2; BPIFB1, BPI fold-containing family B member 1; PDIA3, protein disulfide-isomerase A3; FLOT2, flotillin-2; UBA52, ubiquitin-60S ribosomal protein L40; MSN, moesin; PIGR, polymeric immunoglobulin receptor; ITGB1, integrin beta-1; LGMN, legumain; COLEC12, collectin-12; CTNNB1, catenin beta-1; SFXN1, sideroflexin-1; BCAM, basal cell adhesion molecule; HMOX1, heme oxygenase 1; ANXA3, annexin A3; MYO1C, unconventional myosin-Ic; CTSD, cathepsin D; APOA1, apolipoprotein A-I; DPP4, dipeptidyl peptidase 4; ITGA3, integrin alpha-3; EZR, ezrin; PECAM1, platelet endothelial cell adhesion molecule; FTH1, ferritin heavy chain.

It has been reported that the pathogenesis of pneumonia is closely linked with the dysfunction of the immune system (35, 36). In this project, 53 down-regulated and 32 up-regulated proteins that were implicated in the immune system process were screened out based on the GO annotation analysis. These 85 proteins related to the immune system process were shown in Supplementary Table 11. Among these 85 proteins, 49 proteins that were mapped to the bovine genome were screened out (Supplementary Table 11). Next, the PPI networks of these 49 proteins were established and presented in Figure 6 (organism: Bos taurus, combined interaction score ≥ 0.4) (the solitary proteins have been removed from the network). The detailed protein-protein interaction information and node degrees of the above-mentioned 49 proteins in the PPI networks were displayed in Supplementary Tables 12, 13, respectively. The outcomes suggested that CTNNB1, ITGB1, Annexin A5 (ANXA5), calreticulin (CALR), prothrombin (F2), matrix metalloproteinase-9 (MMP9), platelet endothelial cell adhesion molecule (PECAM1), thrombospondin-1 (THBS1), heat shock protein HSP 90-beta (HSP90AB1), endoplasmin (HSP90B1), integrin alpha-3 (ITGA3), and moesin (MSN) might be the hub proteins in the PPI networks because they had greater node degrees. In other words, these proteins might play vital roles in the immune response related to pneumonia in FMD.

### Validation of differentially expressed proteins by qRT-PCR analysis

**Figure 7:**
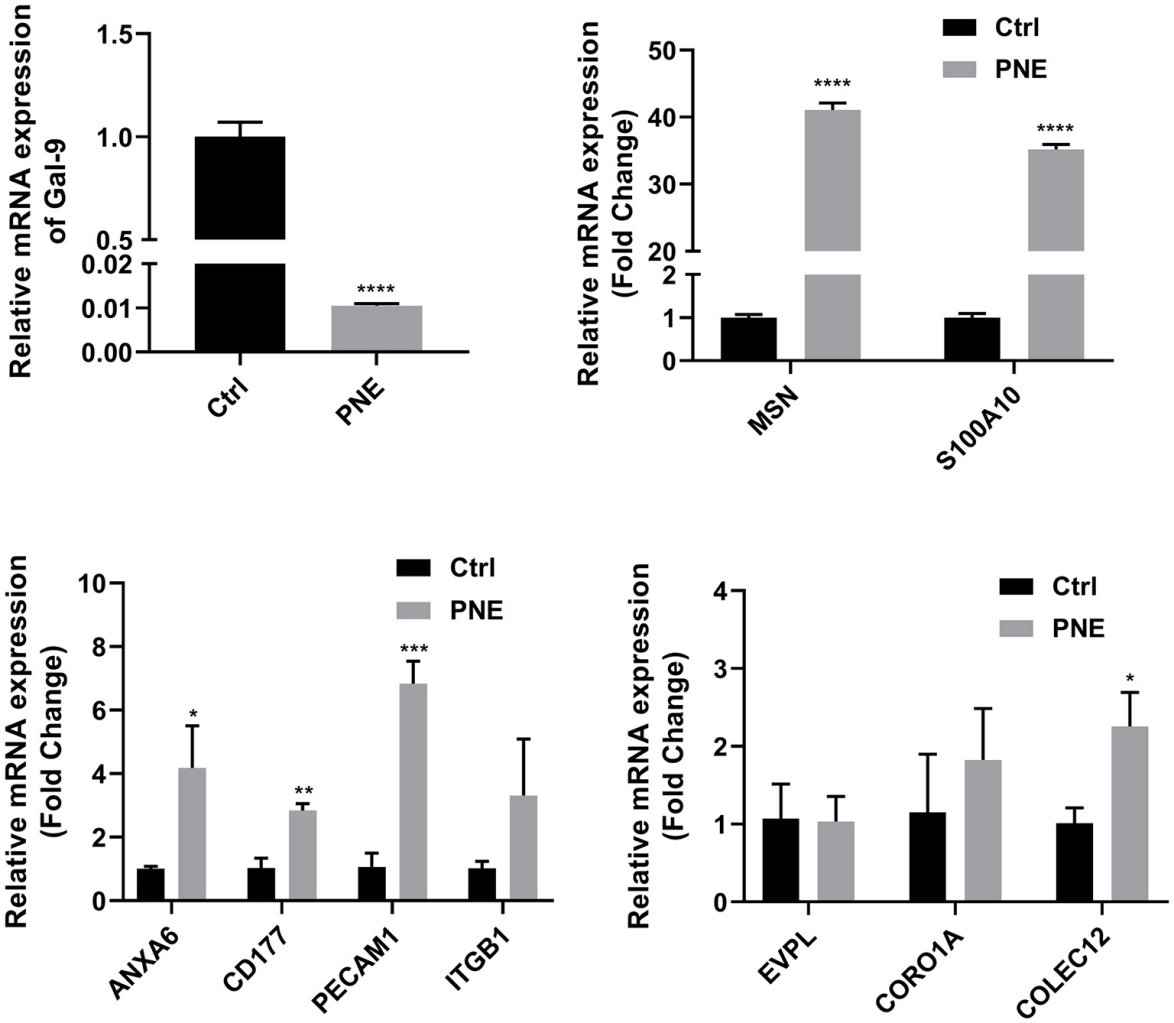
Quantitative RT-PCR analyses of gene expression in normal and diseased tissues. Quantitative expression patterns of genes, which was calculated based on Ct value normalized against the housekeeping GAPDH gene. The *, **, and *** symbols indicate the *P*-value < 0.05, *P*-value < 0.01, and *P*-value < 0.001 respectively.

The expression patterns determined by qRT-PCR were consistent with those obtained by iTRAQ, with 90% agreement between the qRT-PCR and iTRAQ results (Figure 7). This result indicated that the differential proteomic analysis outcomes in this study were reliable.

## Discussion

In this project, a total of 355 differentially expressed (up-regulated ratio ≥ 1.2 or down-regulated ratio ≤ 0.83; adjusted *P*-value < 0.05) proteins were identified in the diseased lung tissues of FMDs who died of pneumonia vs. the normal group. Among these dysregulated proteins, 158 proteins were mapped to the bovine genome.

Moreover, KEGG pathway annotation analysis showed that 9 pathways (i.e., metabolic pathways, focal adhesion, pathways in cancer, regulation of actin cytoskeleton, amoebiasis, microbial metabolism in diverse environments, tight junction, leukocyte transendothelial migration, bacterial invasion of epithelial cells) were shared in the top 20 KEGG pathways of up-regulated and down-regulated proteins. Among the 46 metabolic pathways-related proteins (28 up-regulated and 18 down-regulated), alpha-enolase (ENO1), neutrophil gelatinase-associated lipocalin (LCN2), and Acetyl-CoA acetyltransferase (ACAT1) have been found to be related to pathogens-induced pneumonia. For instance, ENO1 facilitated lipopolysaccharide (LPS)-driven monocyte recruitment to the acutely inflamed lung, and ENO1 was highly expressed in blood monocyte cell surface and alveolar mononuclear cells of patients with pneumonia (37). LCN2 had a potential protective effect against *Escherichia coli*-induced pneumonia (38). LCN2 knockout notably improved the susceptibility of mice to *Acinetobacter baumannii pneumonia* (39). LCN2 hindered the clearance of *pneumococcal pneumonia* and exacerbated pneumococcal pneumonia in mice and humans (40). ACAT1 expression was notably increased in THP-1-derived macrophages following the infection of *Chlamydia pneumonia* (41). The inhibition of ACAT1 weakened pulmonary inflammation and inhibited macrophage activation in bleomycin-induced acute lung injury (42). Among the 27 focal adhesion-related dysregulated proteins (18 up-regulated and 9 down-regulated), thrombospondin-1 (THBS1) and caveolin-1 (CAV1) have been reported to be associated with pneumonia. For example, THBS1 loss promoted the clearance of lung *Klebsiella pneumonia*, decreased lung inflammation burden, and enhanced the innate immune responses against *Klebsiella pneumonia* infection (43). CAV1 depletion reduced mouse survival rate, enhanced bacterial burdens, facilitated bacterial dissemination, and potentiated pro-inflammatory responses in mice infected with *Klebsiella pneumonia* (44). In other words, CAV1 enhanced the resistance of mice to *Klebsiella pneumonia* infection (44). Among 20 proteins related to the regulation of actin cytoskeleton, Rho guanine nucleotide exchange factor 1 (Arhgef1) and myosin light chain kinase (MYLK) have been demonstrated to be related to lung inflammation. For instance, Brown et al. demonstrated that Arhgef1 knockout mice presented decreased airway hyperreactivity and lung inflammation (45). The intravenous injection of MYLK peptide inhibitor reduced lipopolysaccharide-induced lung inflammation in mice (46). Also, 19 (9 down-regulated and 10 up-regulated) and 23 (9 down-regulated and 14 up-regulated) dysregulated proteins in the pneumonia group vs. the control group were identified to be implicated in the amoebiasis and cancer pathways, respectively. As mentioned above, the amoebiasis and cancer pathways were shared in the top 10 KEGG pathways of up-regulated and down-regulated proteins. Although amoebiasis and cancer might be irrelevant or not the study's subject, we supposed that the immunology activity for pneumonia had the same or like response activity in amoebiasis and cancer due to the central roles of pneumonia-induced dysregulated proteins in amoebiasis and cancer. Also, prior studies of some proteins related to amoebiasis and cancer preliminarily validated our speculation. For example, among amoebiasis pathway-related proteins, integrin beta-2 (ITGB2), heat shock protein beta-1 (HSPB1), LCN2, leukocyte elastase inhibitor (SERPINB1), laminin subunit alpha-4 (LAMA4), and fibronectin (FN1) have been found to be correlated with immunity. Wang et al. demonstrated that ITGB2 depletion in combination with CXCR7 and PDGFB knockdown markedly suppressed *Chlamydia pneumonia* entry into human cells (47). Also, ITGB2 has been identified as an immune-related gene (48, 49). HSPB1 inhibitor J2 reduced lung inflammation (50). Epinephelus coioides HSPB1 was a negative regulator in Singapore grouper iridovirus (SGIV)-induced innate immune response and apoptosis (51). LCN2 not only plays a vital role in antibacterial infection but also functions as a crucial player in the immune response to pathogenic inflammatory stimuli (52, 53). SERPINB1 loss increased the susceptibility of mice to pulmonary bacterial and viral infections (54, 55). Also, SERPINB1 controlled neutrophil survival and homeostatic expansion of IL-17+ γδ and CD4+ Th17 cells (56, 57). LAMA4 deficient mice presented impaired recruitment of neutrophils, monocytes, and lymphocytes to inflammatory loci relative to wild-type mice (58). FN1 also has been found to be involved in the regulation of innate immune response and to be correlated with immune infiltrates in cancers (59–61). Among the cancer-related proteins, signal transducer and activator of transcription 3 (STAT3) has been well documented to be inflammation and immunity (62–64). Also, STAT3 served as a positive regulator of pneumonia induced by influenza virus H1N1 (65), *Agiostrongylus cantonensis* (66), and *Mycoplasma pneumonia* (67). Combined with these data, we supposed that dysregulated proteins related to amoebiasis and cancer might also function as crucial players in pneumonia, immune, and inflammation, suggesting the same or like immune response activity between pneumonia and amoebiasis or cancer.

Both KEGG pathway annotation and enrichment analyses showed that dysregulated proteins played vital roles in pathways related to bacterial infection (e.g., bacterial invasion of epithelial cells, *Staphylococcus aureus* infection, and pathogenic *Escherichia coli* infection) and immunity (focal adhesion, phagosome, and complement and coagulation cascades), suggesting that pathways related to bacterial infection and immunity might be closely linked with the development of pneumonia in FMD. Moreover, it has been reported that bacteria including *Staphylococcus aureus* and *Escherichia coli* are common risk factors for pneumonia in FMD (11, 12, 17, 68). Moreover, multiple bacterial pathogens, such as *Leclercia spp., Stenotrophononas maltophila, Staphylococcus aureus*, and *Staphylococcus sciur*, have been identified in bovine pneumonia (69, 70). Thus, differentially expressed proteins in the pathways of *Staphylococcus aureus* infection, bacterial invasion of epithelial cells, and pathogenic *Escherichia coli* infection were screened out. After integration, a total of 32 dysregulated proteins were identified to be implicated in bacterial infection. Among these 32 proteins, 13 proteins, whose sequences were aligned onto the bovine genome, were screened out for further exploration given the close genetic relationships between FMD and bovine. Based on the PPI and node degree analyses of these 13 proteins, we supposed that 5 proteins (CTNNB1, ITGB1, CTNNA1, DNM2, and KRT19) might play crucial roles in bacteria-related pneumonia in FMD. CTNNB1 (protein name: β-catenin) and CTNNA1 are two vital players in the Wnt signaling pathway (71, 72). Wnt/β-catenin signaling has been reported to be a target of bacterial virulence factors (73) and a vital player in lung development and lung diseases (74–77). Additionally, Chen et al. demonstrated that morusin could mitigate mycoplasma pneumonia by inhibiting the Wnt/β-catenin signaling pathway in mice lung tissues (78). ITGB1, also named β1-integrin, hindered bacterial clearance and facilitated bacterial infection in cystic fibrosis airway cells and cystic fibrosis mice (79).

Given the close correlation between immune system dysfunction and pneumonia development, 85 differentially expressed proteins (53 down-regulated and 32 up-regulated) that were implicated in the immune system process were filtered out based on GO annotation analysis. Among these 85 proteins, the sequences of 49 proteins were mapped to the bovine genome. PPI and node degree analysis of these 49 proteins suggested that CTNNB1, ITGB1, ANXA5, CALR, F2, MMP9, PECAM1, THBS1, HSP90AB1, HSP90B1, ITGA3, and MSN might be the hub proteins in the pneumonia-related immune responses in FMD. Some of these proteins have been found to be implicated in pneumonia, lung inflammation, and lung injury. For instance, CALR blockade alleviated acute lung injury (ALI), reduced pro-inflammatory cytokine expression, and inhibited neutrophil and T cell infiltration in bronchoalveolar lavage and lung tissues in lipopolysaccharide (LPS)-induced ALI mouse model (80). MMP9 loss facilitated pulmonary cell death and aggravated lung injury in an interleukin-1β (IL-1β)-induced lung injury mouse model (81). MMP9 acted as a potentially protective factor against *Streptococcus pneumonia* infection (82, 83). PECAM1, an endothelial cell adhesion molecule, played a potentially protective role in lung injury and acute respiratory distress syndrome (84, 85). THBS1 also has been found to be implicated in the pathogenesis of gram-positive bacteria and the development of lung injury (86, 87). For instance, THBS1 loss reduced mouse survival rate, increased lung bacterial burden and lung microvascular permeability, impaired host defense against *Pseudomonas aeruginosa* (*P. aeruginosa*), and potentiated inflammatory injury during *P. aeruginosa* acute intrapulmonary infection (87), while *P. aeruginosa* is a common pathogen of pneumonia in FMD (17). HSP90B1 depletion reduced the phagocytic capacity of macrophages against *Klebsiella pneumonia* (*K. pneumonia)* (a common gram-negative bacteria that can cause pneumonia) and inhibited pro-inflammatory mediator release in alveolar and peritoneal macrophages treated with LPS derived from *K. pneumonia* or heat-killed *K. pneumonia* (88). Moreover, HSP90B1 loss in macrophages led to the increase of mouse lung K. pneumonia loads and are duction in mouse survival rate during *K. pneumonia* (88). CTNNA1 and CTNNB1 are two members of the catenin family (89). Multiple members of catenin family including CTNNA1 and CTNNB1 have been identified to be implicated in immune responses (90–92). For example, CTNNB1 activation enhanced the inflammatory activity of alveolar macrophages and facilitated acute host morbidity in a murine influenza pneumonia model (93). Integrins are crucial players in cell development, cell adhesion, pathogen clearance, inflammation, and immune responses (35, 36). ITGB1 and ITGA3 are two integrin family subunits (94, 95). It has been reported that ITGB1 mediated the entry of coronavirus severe acute respiratory syndrome coronavirus-2 (SARS-CoV-2) (96) and the conditional depletion of ITGB1 in type 2 alveolar epithelial cells could trigger emphysema, epithelial dysfunction, increased efferocytosis and pulmonary macrophage infiltration, and widespread lung inflammation in mice (97). Also, Li et al. suggested that ITGA3 was implicated in the infiltration of 6 immune cells (i.e., B cells, CD8 T cells, CD4 T cells, macrophages, neutrophils, and dendritic cells) in breast cancer (98).

## Conclusions

Taken together, our proteomics analysis revealed that 355 proteins were differentially expressed in diseased lung tissues of FMD that died of pneumonia compared to the normal control group. KEGG annotation and enrichment analysis showed that these dysregulated proteins mainly be associated with bacterial infection and immunity. Moreover, we further screened out the dysregulated proteins related to bacterial infection (*n* = 32) and immunity (*n* = 85). Some key proteins in pneumonia-related bacterial infection and immunity were identified based on PPI and node degree analyses in the FMD. This is the first study to investigate the lung proteomics alterations caused by pneumonia in FMD, which can deepen our understanding of the molecular mechanisms of pneumonia in this rare species. Additionally, the identification of some pathways and proteins that might play vital roles in pneumonia development might contribute to the better management of pneumonia and reduction of mortality rate in FMD. However, only 6 FMD with 3 FMD in each group were used due to the rareness of this species and the difficulty in the acquisition of their organs.

## Data availability statement

The datasets presented in this study can be found in online repositories. The names of the repository/repositories and accession number(s) can be found in the article/Supplementary material. The mass spectrometry proteomics data have been deposited via the iProX partner repository (https://www.iprox.cn/page/home.html) *via* with the dataset identifier PXD031240.

## Author contributions

JT conceived this study, designed, and supervised the experiments. LS, FL, CY, and KB performed the experiments, conducted data analysis, prepared figures and tables. JT wrote the manuscript. YW modified the manuscript. All authors reviewed and approved the manuscript.

## Funding

This research was supported by Science and Technology Program of Shaanxi Academy of Science, China (Program No. 2021K-37) and Shaanxi Province Forestry Science and Technology Innovation Plan (SXLK2021-0222).

## Conflict of interest

The authors declare that the research was conducted in the absence of any commercial or financial relationships that could be construed as a potential conflict of interest.

## Publisher's note

All claims expressed in this article are solely those of the authors and do not necessarily represent those of their affiliated organizations, or those of the publisher, the editors and the reviewers. Any product that may be evaluated in this article, or claim that may be made by its manufacturer, is not guaranteed or endorsed by the publisher.
