# Supplementary material for: ITRAQ-based quantitative proteomics analysis of forest musk deer with pneumonia
Source: Front Vet Sci. 2022 Oct 26;9:1012276. doi: 10.3389/fvets.2022.1012276 (PMC9645242; doi:10.3389/fvets.2022.1012276)
Supplement: Supplementary file 2 [file Data_Sheet_2.docx]

**iTRAQ analysis**

**Sample preparation**

Tissue samples were ground to powder in liquid nitrogen and then lysed using the protein lysis buffer (7M Urea/2M Thiourea/4% SDS/40 mM Tris-HCl (pH 8.5)) supplemented with 1mM PMSF and 2mM EDTA. After incubation for 5 min on ice, 10 mM DTT was added to the samples. The tissue cell suspensions were sonicated for 15 min on ice and then centrifuged at 13,000×g and 4°C for 20 min. The supernatant was mixed with four volumes of -20 °C prechilled acetone and was kept overnight at -20 °C. After centrifugation at 8,000×g and 4 °C for 15min, protein pellets were collected, dried in the air and resuspended in 8 M urea/100 mM triethylammonium bicarbonate (TEAB)(pH 8.0) solution. Next, protein samples were reacted with 10 mM DTT for 30 min at 56˚C and 55 mM iodoacetamide (IAM) for 30 min at room temperature in the dark. Next, protein concentration was determined using the Bradford method.After diluted 5-fold with 100 mM TEAB,proteins (100μg/sample) were digested at 37 °C for 12-16 hourswith trypsinat an enzyme-protein ratio of 1:50 (w/w). The peptide segments post enzymolysis were desalted using C18 columns and thendriedusing the vacuum freezing method.

**iTRAQ labeling and fractionation**

The dried peptides were redissolvedwith 20μlof 0.5M TEAB and labeled following the protocols of iTRAQ Reagent-8 plex Multiplex Kit (SCIEX,Framingham, MA, USA).Samples were labeled using the iTRAQ technology as follows: Phe1, 113;Phe2, 114;Phe3, 117;Ctrl_1, 118;Ctrl_2, 119;Ctrl_3, 121. All of the labeled samples with equal amountwere mixed.After mixed, peptides were fractionated with the increasing concentration of Acetonitrile (ACN) under alkaline condition at the flow rate of 1 ml/min in Durashell C18 column (5 μm, 100Å, 4.6 x 250 mm) on Ultimate 3000high-performance liquid chromatography (HPLC) system (Thermo DINOEX, USA).Distillate fractions were collected per minute. A total of 42 secondary fractions were collected,which was further merged into 12 fractions. The merged fractions were desalted on the Strata-X column and desiccated by vacuum freezing method.

**LC-MS/MS analysis**

The peptide samples were dissolved in 2% acetonitrile/0.1% formic acid and analyzed using TripleTOF 5600+massspectrometry (SCIEX) coupled with an EksigentnanoLC system (SCIEX). A total of 5μg of samples (1μg/μl, 5μl) were added to the C18capture column (5 μm, 100 μm× 20 mm). Samples were eluted at the flow rate of 300 nL/min on C18 analytical columns (3 μm, 75 μm× 150 mm) over a gradient of 90 min. Mobile phase A was composed with 2% acetonitrile, 0.1% formic acid and 98% H_2_O, and mobile phase B was composed with 98% acetonitrile, 0.1% formic acid and 2% H_2_O. For IDA (information dependent acquisition),survey scans were acquired in 250 ms and 30 product ion scans were collected in 50 ms/per scan. MS1 spectrawere collected in the range of 350-1500 m/z, and MS2 spectra were collected in the range of 100-1500 m/z. Precursor ions were excluded from reselection for 15 s.

**Data analysis**

The original MS/MS file data were submitted to Proteinpilot Software v4.5 for data analysis.

The search parameters of Proteinpilot were shown as follows:

Only proteins with at least one unique peptide and unused score ≥ 1.3 (credibility ≥ 95%) were used in the subsequently quantitativeanalysis. The iTRAQquantitative analysisofproteomic data was also carried out using the Proteinpilot Software. The means of pairwise comparisons between biological or technical replicates were firstly normalized as the ratios, and difference between groups was compared using student’s t test.Proteins with a fold changeequalorlarger than 1.2 (i.e.up-regulated ratio ≥ 1.2 or down-regulated ratio ≤ 0.83) and Q value less than 0.05 were considered to be significantly differentially expressed.
